# Systematic failure to operate on colorectal cancer liver metastases in California

**DOI:** 10.1002/cam4.3316

**Published:** 2020-07-20

**Authors:** Mustafa Raoof, Zeljka Jutric, Sidra Haye, Philip H. G. Ituarte, Beiqun Zhao, Gagandeep Singh, Laleh Melstrom, Susanne G. Warner, Bryan Clary, Yuman Fong

**Affiliations:** ^1^ Department of Surgery City of Hope National Medical Center Duarte CA USA; ^2^ Department of Surgery University of California Irvine Irvine CA USA; ^3^ Department of Economics University of California Irvine Irvine CA USA; ^4^ Department of Surgery University of San Diego San Diego CA USA

**Keywords:** disparity, liver metastases, liver resection, population‐level, survival

## Abstract

**Background:**

Despite evidence that liver resection improves survival in patients with colorectal cancer liver metastases (CRCLM) and may be potentially curative, there are no population‐level data examining utilization and predictors of liver resection in the United States.

**Methods:**

This is a population‐based cross‐sectional study. We abstracted data on patients with synchronous CRCLM using California Cancer Registry from 2000 to 2012 and linked the records to the Office of Statewide Health Planning Inpatient Database. Quantum Geographic Information System (QGIS) was used to map liver resection rates to California counties. Patient‐ and hospital‐level predictors were determined using mixed‐effects logistic regression.

**Results:**

Of the 24 828 patients diagnosed with stage‐IV colorectal cancer, 16 382 (70%) had synchronous CRCLM. Overall liver resection rate for synchronous CRCLM was 10% (county resection rates ranging from 0% to 33%) with no improvement over time. There was no correlation between county incidence of synchronous CRCLM and rate of resection (*R* ^2^ = .0005). On multivariable analysis, sociodemographic and treatment‐initiating‐facility characteristics were independently associated with receipt of liver resection after controlling for patient disease‐ and comorbidity‐related factors. For instance, odds of liver resection decreased in patients with black race (OR 0.75 vs white) and Medicaid insurance (OR 0.62 vs private/PPO); but increased with initial treatment at NCI hospital (OR 1.69 vs Non‐NCI hospital), or a high volume (10 + cases/year) (OR 1.40 vs low volume) liver surgery hospital.

**Conclusion:**

In this population‐based study, only 10% of patients with liver metastases underwent liver resection. Furthermore, the study identifies wide variations and significant population‐level disparities in the utilization of liver resection for CRCLM in California.

## Graphical abstract

In this population‐based study, only 10% of patients with liver metastases underwent liver reseaction. Further, the study identifies wide variations and significant population‐level disparities in the utilization of liver resection for CRCLM in California.

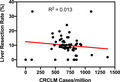

## 1 INTRODUCTION

Colorectal cancer is the second most common cause of cancer deaths in the United States.^1^ At diagnosis, approximately 21% of patients have metastases to other organs. Of these patients, 83% of patients have liver metastases.^2^ Ultimately, 71% of patients with metastatic disease will die as a result of progression of liver metastases.^3^

Liver resection is the only potentially curative therapy for patients with colorectal cancer liver metastases (CRCLM). The utility of liver resection was demonstrated in several landmark observational studies that demonstrated long‐term survival in selected patients with CRCLM.^4^, ^5^, ^6^, ^7^ In fact, in selected patients with liver‐limited disease, liver resection has been shown to result in a median survival of >40 months, with a 5‐year survival of 30%‐55%.^7^, ^8^, ^9^, ^10^, ^11^, ^12^, ^13^, ^14^, ^15^, ^16^, ^17^ In contrast, patients with distant disease undergoing modern systemic chemotherapy achieve a median survival of 24‐30 months.^18^ While individuals with CRCLM rarely (0.5%) survive 10 years with chemotherapy alone,^19^ approximately 20%‐25% of patients survive 10 years or more after liver resection with/without chemotherapy. In fact, those that survive 10 years are now considered cured.^4^, ^8^, ^15^, ^20^

Since a randomized trial to test the benefit of liver resection is not feasible and likely not ethical, an instrumental variable approach—which controls for measured and unmeasured confounders—was used to demonstrate the causal effect of liver resection on patient survival.^21^ These results show that for patients in whom the liver resection was influenced by their geographic area of residence (marginal patients), increasing rate of liver resection of their geographic residence area would have significantly and positively impacted survival.^21^ These observations provide a strong rationale for liver resection as life‐prolonging and potentially curative therapy. While liver resection has now become routine practice in tertiary centers across the United States,^22^, ^23^, ^24^, ^25^, ^26^, ^27^, ^28^ the population‐level data for utilization of liver resection in the United States are lacking. There is a growing body of literature that supports the notion that hepatectomy is underutilized. In a recent publication, it was noted that California Medical Service Study Area hepatectomy rates ranged from 2.7% (lowest quintile) to 19.2% (highest quintile).^21^ This finding also appears to be present even among patients being treated in academic centers in the confines of clinical trials where hepatectomy rates in previously irresectable patients receiving contemporary systemic therapy varies from single digits to over 60%.^29^ Given the growing disparities in access to complex surgical care in the United States, we hypothesized that liver resection use will be associated with patient's sociodemographic and health‐system‐related factors.

The purpose of this study is to characterize variation in liver resection utilization rates in California. In addition, we examine associations between liver resection utilization and geospatial, temporal, patient, and hospital factors in a population‐based cohort of patients. Finally, we provide population‐level estimates of overall and disease‐specific survival in patients with CRCLM with or without liver resection.

## 2 METHODS

### 2.1 Databases

The California Cancer Registry (CCR) is one of the most complete cancer registries in the country.^30^ In California, reporting of cancer care is mandatory, yielding high rates of patient capture and subsequent follow‐up. Patient discharge data (PDD) after inpatient hospitalization were acquired from the California Office of Statewide Health, Planning, and Development (OSHPD). The PDD files contain patient‐level data for all general, acute‐care, non‐federal hospitals in California. For each admission, the PDD files include principal diagnosis and as many as 24 secondary diagnoses coded using the *International Classification of Diseases, Ninth Revision, Clinical Modification* (*ICD‐CM‐9*) format, the principal procedure, and as many as 20 secondary procedures. This information enables a more accurate assessment of patient comorbidities and more detailed information on surgical procedures than is currently available from any cancer registry data alone. To calculate incidence rates, the county‐level population data were obtained from RAND State Statistics (RAND Corporation, Santa Monica, CA). This database estimates total resident population on July 1st of each year after adjustments for the United States, states, counties, and places (also known as cities or towns). The benchmark for population estimates is the 2000 Census of Population and Housing. The study was approved by the state and institutional review boards, with a waiver of informed consent.

### 2.2 Database linkage

Cases identified in the CCR from 1 January 2000 through 31 December 2012 were linked to PDD files from OSHPD by applying a probabilistic linking algorithm based on sex, date of birth, and social security number as described previously.^31^ Follow‐up data were available through 31 December 2015.

### 2.3 Study cohort

#### 2.3.1 Patient eligibility and exclusion criteria

We included patients from CCR with histologically confirmed colorectal cancers based on ICD‐O‐3 codes for site (C180, C182‐C189, C199, and C209) and histology codes for adenocarcinoma (814, 821, 822, 848, 849, and 857). Patients with synchronous liver metastases (CRCLM within 6 months of diagnosis) at diagnosis were identified from CCR. These cases were cross‐referenced to data obtained from PDD. Since CCR does not include patients with metachronous liver metastases, these patients were excluded from the study cohort. We excluded cases that were without histologically confirmed diagnoses, with other primary malignancies, <18 years of age, diagnosed at autopsy, diagnosed in hospice, or without follow‐up information. While presence of extrahepatic metastases was once considered an absolute contraindication to liver resection, recent literature challenges this assertion.^32^ Therefore, patients with extrahepatic liver metastases were not excluded from analysis. The specific exclusion steps are presented in the Supplementary Information (Table S1).

#### 2.3.2 Patient's data variables and definitions

Variables included age, race/ethnicity, socioeconomic status, insurance, distance traveled to the treatment facility, sex, marital status, Charlson‐Deyo score^33^ for comorbid conditions, tumor laterality, grade, nodal status, extrahepatic metastases, resection of primary and receipt of chemotherapy. As almost all liver resections require inpatient hospitalization, performance of liver resection was confirmed with ICD‐9‐CM procedure codes for liver resection in PDD files: 50.22, 50.3, and 50.4. Socioeconomic index was defined based on Yost‐Yang socioeconomic index.^34^ Briefly, this is an individual‐level socioeconomic status approximation based on census block characteristics derived from residential address, ranked and reported as quintiles. Distance between patient residence and hospital was calculated by using latitude and longitude of zip code centroids, and then applying the Haversine formula to calculate the great circle distance in miles between these two points.^35^ The distance of 0 miles would indicate patient and hospital were located in the same zip code.

#### 2.3.3 Hospital‐level variables and definitions

CCR records identify the facility reporting data to the registry. For 95% of patients in this study cohort, the reporting facility was where the first course of treatment was initiated. Hereafter, the facility where the treatment was initiated is referred to as the treatment‐initiating‐facility (TIF). A TIF was classified as a teaching hospital or not based on 2010‐2012 healthcare facilities descriptive data files maintained by OSHPD. Hospital location (urban and rural) was extracted from OSHPD data files. We classified TIF as a safety‐net hospital as those represented in the top quartile of Medicaid or uninsured admissions,^36^ based on all CA state acute care facilities. National Cancer Institute (NCI) designated cancer center was identified through the NCI “Find a Cancer Center.”^37^ For the purposes of the study, if a hospital performed at least one liver resection for colorectal liver metastases, it was classified as a liver surgery hospital, whereas if a hospital performed 10 or more liver resections per year, it was classified as a high volume liver surgery hospital. The cut‐off of 10 was based on the analysis of the Nationwide Inpatients Sample by Dimick et al.^38^

#### 2.3.4 Outcome

The primary outcome was liver resection rate in patients with synchronous CRCLM in California. The secondary outcomes were as follows: receipt of liver resection, overall and disease‐specific survival probabilities.

### 2.4 Statistical analyses

#### 2.4.1 Data reporting

Categorical variables are presented as frequencies with percentages. Continuous variables such as distance and age are classified based on quartiles. Univariate analyses are performed using chi‐square test.

#### 2.4.2 Statistical models

The selection of variables in the multivariable models was theory driven and was further informed by results from univariate analysis. To determine patient‐level predictors of liver resection, we used a mixed‐effects (multi‐level) logistic regression model (melogit command in Stata). A mixed‐effects model was used because the data were considered correlated (patients treated at the same TIF were potentially similar in terms of the care they received and their eventual outcome). We confirmed that the mixed‐effects model was superior to a conventional (fixed‐effects) logistic regression model using a likelihood ratio test (*P* < .0001). Confidence intervals of effect sizes are estimated using robust standard errors. In additional analyses, patient county of residence was added as a fixed effect. To determine hospital‐level predictors of liver resection, we modeled the data using a fixed‐effects logistic regression (logistic command in Stata) while adjusting for patient‐level variables. In these models, we report confidence interval of effect sizes using clustered standard errors to account for hierarchical nature of the data. Because of concerns for multicollinearity, each hospital characteristic was modeled separately. To account for clustering by patient's county of residence, a mixed‐effects logistic regression model was used where county was set as a fixed effect.

Survival curves are constructed using the Kaplan‐Meier method. Follow‐up was measured from the time of diagnosis to date of death or last contact. For overall survival, a failure event was defined as any death. Those alive at last follow‐up were censored. For disease‐specific survival, a failure was defined as death due to colorectal cancer. Those that died of another cause or those that were alive at last follow‐up were censored. The survival function was compared using a log‐rank test.

For all statistical analyses, we used Stata/MP software (version 14.1; StataCorp LLP) with assumption of two‐sided tests and a criterion for statistical significance set at *α* < .05 unless otherwise indicated. Geospatial mapping was performed using Quantum Geographic Information System (version 3.6).

## 3 RESULTS

### 3.1 Utilization of liver resection in California

**FIGURE 1:**
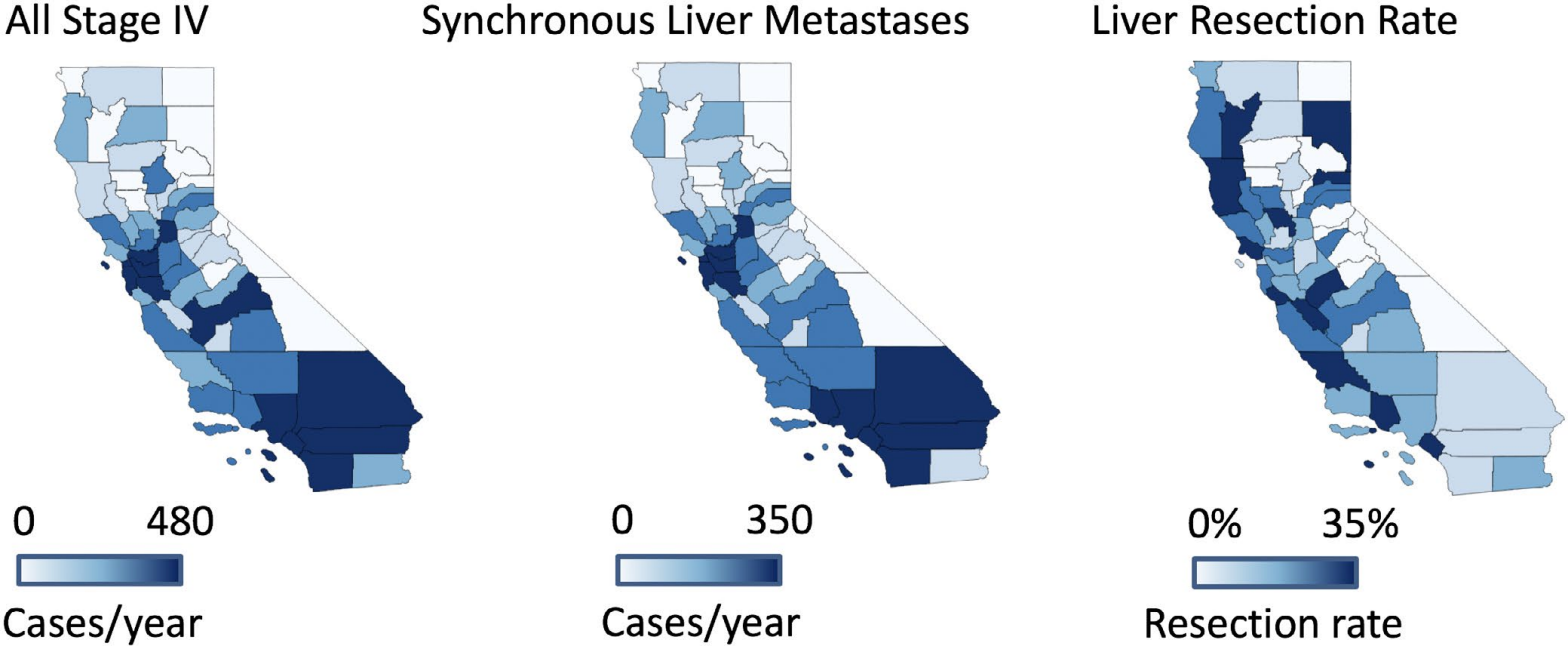
County‐level variation in the utilization of liver resection in California

**FIGURE 2:**
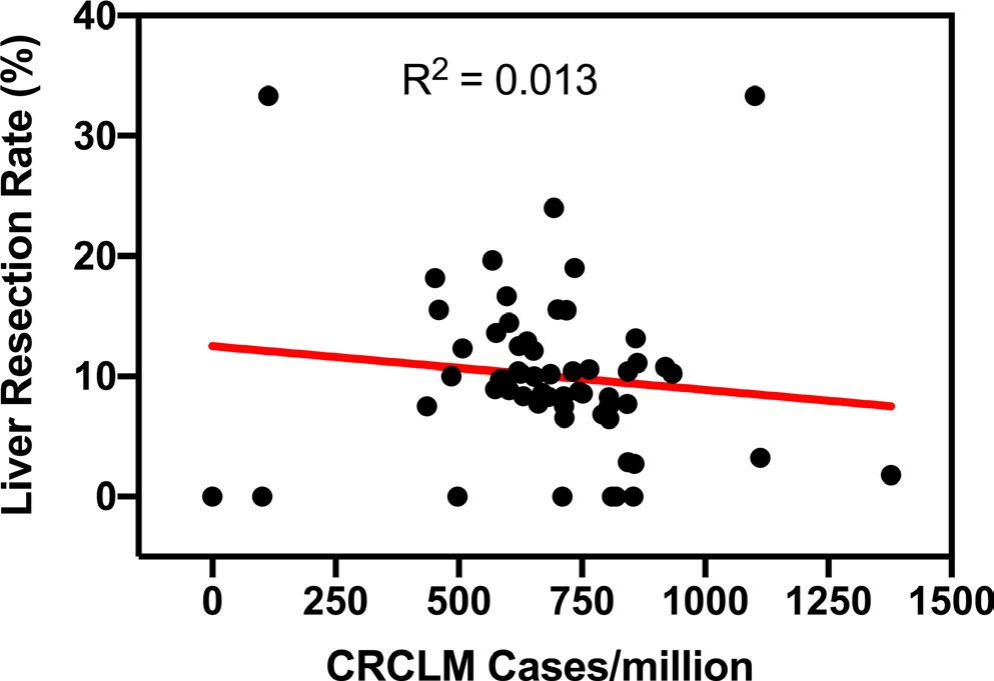
Lack of correlation between annual county‐level incidence of synchronous colorectal cancer liver metastases and liver resection rate in California

Of the 24 828 patients diagnosed with stage‐IV colorectal cancer, 16 382 (70%) had synchronous CRCLM. Overall liver resection rate for synchronous CRCLM was 10% (County resection rate range 0%‐33%). Among patients with liver limited disease, the liver resection rate was 13% (861/6370). County‐level geospatial distribution of occurrence of stage‐IV colorectal cancer, those with liver metastases and those undergoing resection are shown in Figure 1. Annual cases of all stage‐IV colorectal cancer averaged over the duration of study period in each county ranged from 0 to 480. During the same time, the occurrence of synchronous liver metastases in each county ranged from 0 to 344 cases per year. As shown in Figure 2, there was no correlation between the incidence of CRCLM and receipt of liver resection for a given county.

### 3.2 Temporal trends in liver resection utilization

**FIGURE 3:**
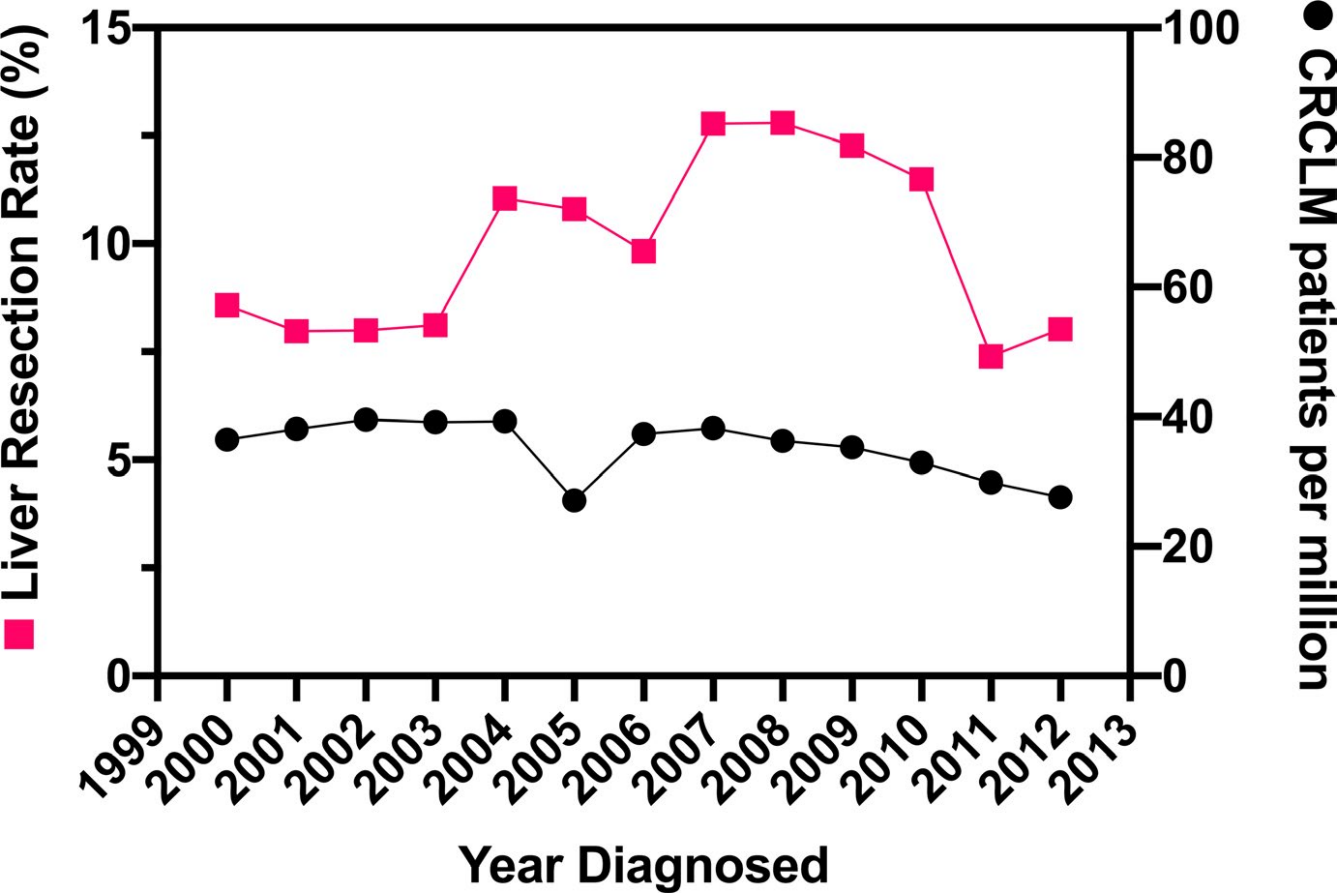
Temporal trends in the annual state‐level incidence of synchronous colorectal cancer liver metastases and liver resection rate in California

Temporal trends are demonstrated in Figure 3. As shown, the incidence of CRCLM has declined overtime from 3.6 cases/100 000 in 2000 to 2.8 cases/100 000 in 2012. The rate of liver resection fluctuated from 8.0% to 12.8% during this time. There was a period of increased liver resection utilization from 2004 to 2010 which may be attributed to FDA approval of oxaliplatin, irinotecan, and biologics during this time. However, there was a decrease in liver resection rates in 2011 and 2012 for unclear reasons.

### 3.3 Demographic and clinicopathologic characteristics

Patient demographic and clinicopathologic data are summarized in Table 1. As shown, patients undergoing liver resection tended to be younger, with fewer comorbidities, married, with higher‐socioeconomic status, and have private insurance. They were also more likely to travel farther to receive treatment, be of non‐minority race/ethnicity. In terms of disease characteristics, those who received liver resection had less frequent extrahepatic metastases, lower grade tumors, and left‐sided tumors. Furthermore, receipt of primary site resection and/or peri‐operative chemotherapy was associated with receipt of liver resection.

**TABLE 1:** Demographic, clinicopathologic, and treatment characteristics of the study cohort

Characteristic	N = 16 382	Liver resection	No liver resection	*P*‐value
		n (%)	n (%)	
Age (years)	18‐55	656 (40)	3487 (24)	<.001
	56‐66	525 (32)	3771 (26)	
	67‐76	318 (19)	3621 (25)	
	77‐103	136 (8)	3868 (26)	
Sex	Male	870 (53)	7834 (53)	.946
	Female	765 (47)	6913 (47)	
Comorbidities	None	1386 (85)	10 908 (74)	<.001
	One	210 (13)	2691 (18)	
	Two+	39 (2)	1148 (8)	
Marital status	Not married	552 (34)	7076 (48)	<.001
	Married	1083 (66)	7671 (52)	
Year of diagnosis	2000‐2003	431 (26)	4850 (33)	<.001
	2004‐2008	736 (45)	5666 (38)	
	2009‐2012	468 (29)	4231 (29)	
Socioeconomic status	Very low	174 (11)	2405 (16)	<.001
	Low	248 (15)	2883 (20)	
	Middle	286 (17)	3106 (21)	
	High	404 (25)	3099 (21)	
	Very high	453 (28)	2682 (18)	
Race/ethnicity	White	997 (61)	8667 (59)	<.001
	Black	98 (6)	1493 (10)	
	Hispanic	257 (16)	2463 (17)	
	Asian/PI	223 (14)	1763 (12)	
	Middle Eastern	50 (3)	268 (2)	
Insurance	Private/PPO	735 (45)	5038 (34)	<.001
	HMO	249 (15)	1669 (11)	
	Medicare	451 (28)	5660 (38)	
	Medicaid/indig	160 (10)	2097 (14)	
	Federal	6 (<1)	37 (<1)	
Distance traveled (miles)	0‐3	342 (21)	3676 (25)	<.001
	3.1‐5.9	372 (23)	3646 (25)	
	6.0‐11.2	417 (26)	3607 (25)	
	>11.3	474 (29)	3537 (24)	
Grade	Low	68 (4)	574 (4)	<.001
	Moderate	1164 (71)	7837 (53)	
	Poor	333 (20)	3629 (25)	
	Undifferentiated	14 (1)	222 (1)	
Node positive	No	582 (36)	7516 (51)	<.001
	Yes	1053 (64)	7231 (49)	
Extrahepatic metastases	No	861 (53)	6370 (43)	<.001
	Yes	774 (47)	8377 (57)	
Primary ‐site	Right colon	569 (35)	6000 (41)	<.001
	Left colon/rectum	1066 (65)	8747 (59)	
Primary ‐ resection	No	58 (3)	58 (3)	<.001
	Yes	1577 (96)	9352 (63)	
Chemotherapy	No	275 (17)	6613 (45)	<.001
	Yes	1321 (81)	7566 (51)	

### 3.4 Patient‐level predictors of liver resection

Results from multivariable mixed‐effects logistic regression analysis are summarized in Table 2. In this model, patients are clustered by treatment initiating facility, that is, we account for the fact that patients treated at a given TIF tend to be treated similarly. After adjusting for TIF, the model demonstrates that odds of liver resection decreased with age (OR 0.97/year), two or more comorbidities (OR 0.5 vs 0 comorbidities), black race (OR 0.75 vs white), mediacid insurance (OR 0.62 vs private/PPO), poorly differentiated tumors (OR 0.6 vs well‐differentiated tumors), or extra‐hepatic metastases (OR 0.57). Odds of liver resection increased with being married (OR 1.27), having very high socioeconomic status (OR 1.65 vs very low), left‐sided tumors (OR 1.22), primary tumor resection (OR 14.91), or peri‐operative chemotherapy (OR 2.51). When we repeated these analyses by additionally accounting for clustering of patients based on their county of residence, we observed similar results (Table S2).

**TABLE 2:** Multivariable analysis ‐ predictors of receipt of liver resection

Characteristic	N = 16 382	Odds ratio	[95% Conf. Interval]		*P*‐value
Age (years)	**(Continuous)**	**0.97**	**0.97**	**0.98**	**<.001**
Comorbidities	None	1 (base)			
	One	0.90	0.77	1.05	.178
	**Two+**	**0.50**	**0.36**	**0.69**	**<.001**
Marital status	Not Married	1 (base)			
	**Married**	**1.27**	**1.12**	**1.44**	**<.001**
Year of diagnosis	2000‐2003	1 (base)			
	**2004‐2008**	**1.41**	**1.20**	**1.66**	**<.001**
	**2009‐2012**	**1.25**	**1.05**	**1.50**	**.013**
Socioeconomic status	Very Low	1 (base)			
	Low	1.13	0.90	1.41	.290
	Middle	1.07	0.84	1.35	.593
	**High**	**1.48**	**1.20**	**1.84**	**<.001**
	**Very High**	**1.65**	**1.30**	**2.10**	**<.001**
Race/ethnicity	White	1 (base)			
	**Black**	**0.75**	**0.58**	**0.95**	**.020**
	Hispanic	0.93	0.79	1.10	.429
	Asian/PI	1.02	0.85	1.22	.805
	Middle Eastern	1.35	0.93	1.94	.110
Insurance	Private/PPO	1 (base)			
	HMO	1.03	0.85	1.24	.777
	Medicare	1.03	0.88	1.22	.677
	**Medicaid/indig**	**0.62**	**0.51**	**0.76**	**<.001**
	Federal	1.07	0.43	2.66	.878
Grade	Well	1 (base)			
	Moderate	0.95	0.72	1.27	.742
	**Poor**	**0.60**	**0.44**	**0.81**	**.001**
	**Undifferentiated**	**0.38**	**0.20**	**0.72**	**.003**
Extrahepatic metastases	No	1 (base)			
	**Yes**	**0.57**	**0.51**	**0.65**	**<.001**
Primary ‐site	Right Colon	1 (base)			
	**Left Colon/Rectum**	**1.22**	**1.10**	**1.35**	**<.001**
Primary ‐ resection	No	1 (base)			
	**Yes**	**14.91**	**11.15**	**19.90**	**<.001**
Chemotherapy	No	1 (base)			
	**Yes**	**2.51**	**2.19**	**2.87**	**<.001**

Note

Results from multivariate mixed‐effects logistic regression model are demonstrated with patients clustered by treatment initiating facility.

Bolded text indicates statistically significant values (ie *P* < .05).

### 3.5 Hospital‐level predictors of liver resection

Liver resection rates by region and TIF characteristics are summarized in Tables S3 and S4. Results from multivariable logistic regression analyses evaluating TIF characteristics are summarized in Table 3. One TIF characteristic was evaluated per multivariable logistic regression model. These models accounted for patient sociodemographic and clinicopathologic characteristics. Because several TIF characteristics were collinear, an all‐encompassing model with all TIF characteristics was not possible. As shown, the odds of liver resection were significantly higher if the TIF was an NCI‐designated cancer center (OR 1.69) or a high volume (10 + cases/year) liver surgery center (OR 1.40). Teaching hospital status, safety‐net hospital status, location of the hospital (urban vs rural), or liver surgery center status was not associated with the receipt of liver resection. We repeated these analyses but this time modeling the data as being nested by patient's county of residence. Results shown in Table S5 are found to be robust to patient's county of residence.

**TABLE 3:** Multivariable analysis ‐ hospital‐level predictors of liver resection

Characteristic	N = 16 382	Odds Ratio	[95% Conf. Interval]		*P*‐value
Teaching Hospital					
	No	1 (base)			
	Yes	1.23	0.97	1.57	.091
Hospital Location					
	Urban	1 (base)			
	Large rural	0.92	0.66	1.27	.604
	Small rural	1.04	0.70	1.54	.849
Safety‐net hospital					
	No	1 (base)			
	Yes	0.92	0.74	1.14	.459
Liver surgery hospital (at least 1 liver resection performed)					
	No	1 (base)			
	Yes	0.96	0.81	1.13	.616
High volume liver surgery hospital (10 + liver resections performed/year)					
	No	1 (base)			
	**Yes**	**1.40**	**1.15**	**1.67**	**.001**
NCI Cancer Center					
	No	1 (base)			
	**Yes**	**1.69**	**1.22**	**2.36**	**.002**

Note

Multivariate logistic regression models adjusted for patient characteristics (age, marital status, comorbidities, year of diagnosis, type of insurance, race/ethnicity, socioeconomic status, primary site of tumor, grade of tumor, resection of primary tumor, peri‐operative chemotherapy, and extrahepatic metastases). For each hospital characteristic, a separate multivariable model was developed. Estimates are based on clustered standard errors.

Bolded text indicates statistically significant values (ie *P* < .05).

### 3.6 Population‐level survival estimates after liver resection for CRCLM

**FIGURE 4:**
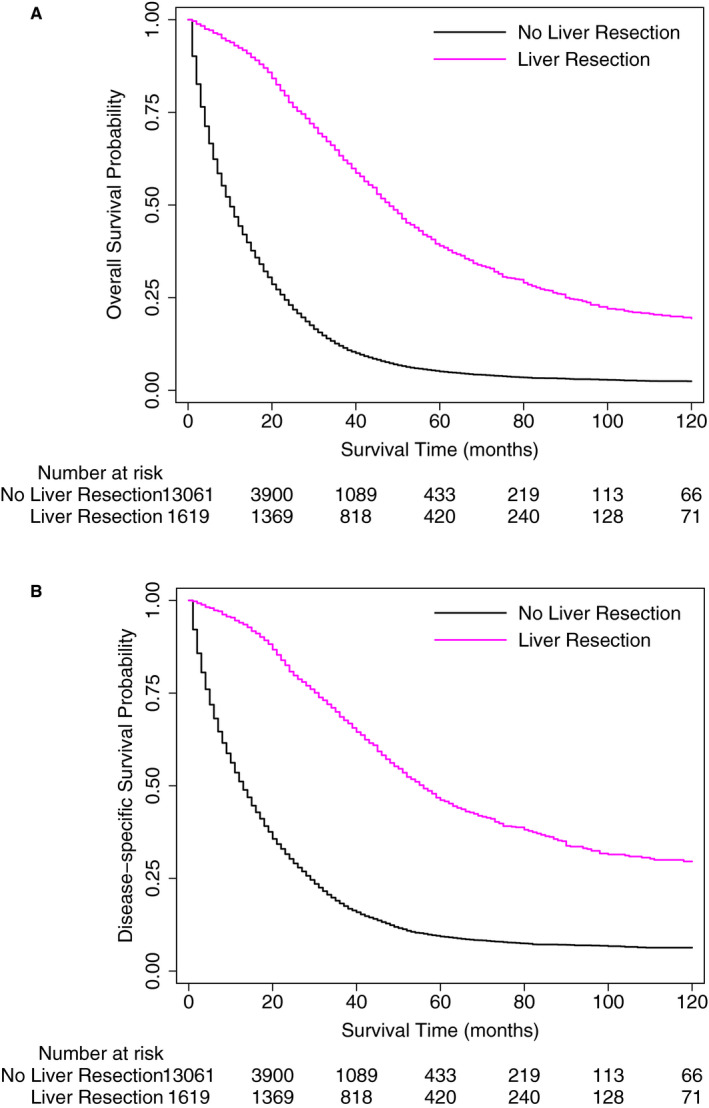
Kaplan‐Meier Survival Analysis depicting overall (A) and disease‐specific (B) survival in synchronous colorectal cancer liver metastases patients stratified by receipt of liver resection. Log‐rank test *P* < .0001 for both comparisons

Kaplan‐Meier Survival estimates stratified by receipt of liver resection are shown in Figure 4. As shown, patients with synchronous CRCLM undergoing liver resection had an improved overall and disease‐specific survival compared to those patients that did not undergo liver resection. Log‐rank *P* value for both comparisons <.0001.

The median overall survival in patients undergoing liver resection was 48 months in comparison to 10 months in those who did not undergo liver resection, resulting in a 10‐year overall survival of 19% vs 2%, respectively. Similarly, the median disease‐specific survival in patients undergoing liver resection was 55 months in comparison to 12.5 months in those who did not undergo liver resection, resulting in a 10‐year disease‐specific survival of 30% vs 6%, respectively.

## 4 DISCUSSION

Liver resection is a life‐prolonging and potentially curative treatment for well‐selected patients with CRCLM. There is a very large body of literature comprising single institutional series and multi‐institutional retrospective analyses from expert centers that supports this assertion.^2^, ^3^, ^4^, ^5^, ^6^, ^7^, ^8^, ^9^, ^10^, ^11^, ^12^, ^13^, ^14^, ^15^, ^16^, ^17^, ^20^, ^22^, ^23^, ^24^, ^25^, ^26^, ^27^, ^28^, ^32^ A recent instrumental variable analysis provides causal estimates of improved survival after liver resection in a population‐based cohort.^21^

Utilization of liver resection and factors associated with receipt of liver resection for CRCLM in the US population remain unknown. Population‐level cancer registries (such as NCI Surveillance Epidemiology and End Results) do not capture utilization of liver resection for metastatic disease. Commonly used hospital‐level databases (such as National Cancer Database, Multi‐institutional collaborations, Nationwide Inpatient Sample, and American College of Surgeon's NSQIP database) do no capture data regarding patients that are not hospitalized. By linking data from patient discharge records from OSHPD to those from California Cancer Registry, this study provides real‐world population‐level estimates of utilization of liver resection in a population‐based cohort. Furthermore, this study identifies several barriers to the use of liver resection in the context of the US healthcare system.

The main finding of the study is that only 10% of patients with synchronous CRCLM undergo liver resection in California. Historically, at expert centers in the United States, this rate was 20%.^39^ More recenty, with judicious use of conversion chemotherapy, at least 12%‐33% of patients with initially unresectable disease will become candidates for resection with expectations of excellent outcomes.^40^, ^41^ Population‐based studies from other countries demonstrate overall liver resection rate to be as follows: 3%‐4% in Canada^24^, ^26^; 4%‐18% in Sweden^42^, ^43^, ^44^; 13% in Australia^45^; 19% in Germany^46^; and 2%‐26% in Netherlands.^27^, ^47^ In light of prior literature, the results demonstrate that the utilization of liver resection in California is very low. Our analysis also demonstrates that while the incidence of synchronous CRCLM only declined slightly, there has been no increase in the overall use of liver resection as would be expected based on its utility in improving survival. Finally, we found that while there was variation in the utilization of liver resection among counties (0%‐33%), there was no correlation between the incidence of synchronous CRCLM and liver resection rate of the county. This finding points to a systematic failure to direct appropriate patients for liver resection. Alternatively, this lack of correlation could be explained by migration of patients across counties. However, we find that the majority (~75%) of individuals with synchronous CRCLM started their first course treatment within 11 miles of their residence. Furthermore, complex multi‐level models accounting for patient county of residence yielded similar results suggesting that this was not a major factor in the utilization of liver resection.

To test the hypothesis if sociodemographic factors impact the receipt of liver resection, we performed univariable and multivariable analyses. The results demonstrate that after accounting for measurable patient disease characteristics and accounting for TIF clustering, there were significant imbalances in the sociodemographic profiles of patients who receive liver resection. Race and insurance‐based disparities have previously been identified in the context of nationwide analysis of hospitalized patients with CRCLM, some of whom underwent liver resection.^48^ However, restricting analysis to a hospital‐based cohort can under‐represent individuals who are marginalized. The present study overcame these limitations and identified several additional independent sociodemographic predictors of failure to receive liver resection including advanced age, black race, Medicaid insurance, unmarried status, and low socioeconomic status.

The hospital that initiates first‐course treatment is likely an important determinant in receipt of liver resection. To test this hypothesis, we identified hospital‐level predictors of the receipt of liver resection after accounting for patient‐level factors. Our results indicated that patients were more likely to get liver resection if the TIF was an NCI‐designated cancer center or a high volume liver surgery center. Patients with CRCLM are most frequently encountered by medical oncologists. While at least 85% of medical oncologists participate in multidisciplinary tumor boards^49^, the representation of liver surgeons in these tumor boards is likely low. For instance, Krell et al surveyed medical oncologists in Michigan and identified that 40% of respondents did not have access to liver surgeons in their practice area.^50^ In this study, the authors also found that while medical oncologists can correctly refer low recurrence risk CRCLM patients to liver surgeons, there was a significant underestimation of resectability of moderate and high‐recurrence risk patients. These observations underscore the need to improve accessibility of liver surgeons in community oncology tumor boards. Consistent with this argument, the present analysis demonstrates that patients who receive initial care at a facility that does not routinely perform liver resection are much less likely get a liver resection. While the reason for higher odds of liver resection at NCI‐designated cancer centers or high‐volume liver surgery centers is not entirely clear from the present analysis, we speculate that participation of liver surgeons in the multidisciplinary care of patients with CRCLM will likely improve the utilization of liver resection for appropriate patients.

This study provides the first population‐level estimates of survival in those who did and did not undergo liver resection in California. The patients in the two groups are in no way comparable and the improved survival in the liver resection group cannot be directly attributed to liver resection. Instead, these estimates provide real‐world baseline survival statistics for a US population‐based cohort. Taken together, the underutilization and disparities in the use of liver resection reported in this study and the population‐level causal estimates of survival benefit of liver resection demonstrated in recent work^21^; these data provide a strong rationale to affect health policy change.

Despite the strengths of these analyses, there are several limitations inherent to the study's retrospective design. The models developed in this study provide correlative evidence and do not account for unmeasured confounders which may impact the effect size of our estimates. The study does not account for metastatic burden of disease within the liver; and hence biologic and technical resectability. It is possible that patients with synchronous CRCLM in California are more likely to have unresectable disease in comparison to that reported in institutional studies and foreign population‐based analyses. However, this is unlikely because in our analysis we found a large variation in liver resection rate that did not correlate with the incidence of synchronous CRCLM suggesting other factors are important. The authors acknowledge that the rates of liver resection for metachronous liver metastases could be higher and were not investigated in this study. In California, metachronous liver resections comprise only one‐third of all liver resections for CRCLM (data not shown). Therefore, despite this limitation the study captures the majority of liver resections in California. While the estimates are population‐based, they may not be generalizable to rest of the United States. For instance, California is healthier (Ranked 12 based on 35 core health‐related measures) than most states in the United States and our results may over‐estimate liver resection utilization and under‐estimate disparities in the United States.^51^

In conclusion, this is the first study to characterize the striking underuse of hepatectomy for CRCLM in California at a population level. Moreover, we have identified the strong influence of treatment initiating facility on the receipt of liver resection. These results highlight a systematic failure and have implications for directing health policies that increase liver resection rates to improve survival of patients with CRCLM. Improving general access to affordable health care for marginalized patients will likely improve the utilization of treatment for CRCLM inclusive of liver resection. Beyond general improvements in healthcare access, next steps must include raising awareness of the utility of liver resection through community outreach especially among medical oncology practices and perhaps those of colorectal and general surgeons who are also often frontline providers for synchronous patients. National Quality Forum and Commission on Cancer can propose a national benchmark that endorses evaluation of each CRCLM patient by an accredited liver surgeon. Privatized Medicare and other insurance plans must include and allow access to liver surgeons in their network. Finally, cancer registries should include more granular information on the burden of liver metastases in patients with CRCLM to track progress going forward. The present study lays the foundation for future work focusing on reducing disparities in liver resection utilization for CRCLM.

## CONFLICT OF INTEREST

None.

## AUTHORS’ CONTRIBUTIONS

Conceptualization: MR, ZJ, SGW, BC, and YF; Data curation: MR, ZJ, and PHGI; Formal Analysis, Investigation, Methodology, and Project Administration: MR, ZJ, SH, and PHGI; Funding acquisition and Resources: NA; Software: MR, SH, and PHGI; Supervision: BC and YF; Validation, Visualization, and Writing—original draft: MR, SH, and PHGI; Writing—review and editing: All authors.

## Supporting information

Table S1‐S5

## Data Availability

The data that support the findings of this study are available from California Cancer Registry and Office of Statewide Health Planning. Restrictions apply to the availability of these data, which were used under license for this study.
