# Supplementary material for: Systematic failure to operate on colorectal cancer liver metastases in California
Source: Cancer Med. 2020 Jul 20;9(17):6256–67. doi: 10.1002/cam4.3316 (PMC7476837; doi:10.1002/cam4.3316)
Supplement: Supplementary file 1 — Table S1‐S5 [file CAM4-9-6256-s001.docx]

**Supplemental Information**

**Systematic Failure to Operate on Colorectal Cancer Liver Metastases in California**

**Table S1**. **Flowchart describing** **Inclusion and Exclusion of Patients in the Study**

| Step | Criteria | Total | Total Pts Left |
| --- | --- | --- | --- |
| 0 | All California Cancer Registry cases 2000-2012 | --- | 1,541,480 |
| 1 | Exclude sites other than colorectal cancers | 1,363,487 | 177,993 |
| 2 | Exclude non-analytic cases | 4,368 | 173,625 |
| 3 | Exclude patients who did not have a histologically confirmed diagnosis | 2,961 | 170,664 |
| 4 | Exclude if CRC was not the only or first primary cancer diagnosis | 29,605 | 141,059 |
| 5 | Exclude non-adults (< 18 yo) | 31 | 141,028 |
| 6 | Exclude patients diagnosed by autopsy/death record | 0 | 141,028 |
| 7 | Exclude patients diagnosed in nursing home/hospice | 9 | 141,019 |
| 8 | Exclude if no diagnosis date | 641 | 140,378 |
| 9 | Exclude if no follow-up date | 29 | 140,349 |
| 10 | Exclude patients if surgical details were unknown | 36 | 140,313 |
| 11 | Exclude cases that were unmatched to patient discharge data | 9,781 | 130,532 |
| 12 | Exclude cases with mets diagnosis date more than 6 months before colorectal cancer diagnosis (Coding Error) | 7 | 130,525 |
| 13 | Excluding cases that were not AJCC 6^th^ edition Stage IV with liver mets (ICD9 code 197.7) | 105,697 | 24,828 |
| 14 | Exclude cases with metachronous liver mets (> 6 months after colorectal cancer diagnosis date in CCR | 8,446 | **16,382** |

**SUPPLEMENTAL ANALYSIS**
